# Comparative Analysis of Vaccine-Induced Neutralizing Antibodies against the Alpha, Beta, Delta, and Omicron Variants of SARS-CoV-2

**DOI:** 10.3390/vaccines12050515

**Published:** 2024-05-09

**Authors:** Philipp Girl, Heiner von Buttlar, Enrico Mantel, Markus H. Antwerpen, Roman Wölfel, Katharina Müller

**Affiliations:** 1 Bundeswehr Institute of Microbiology, 80937 Munich, Germany; philippgirl@bundeswehr.org (P.G.); heinervonbuttlar@bundeswehr.org (H.v.B.); enricomantel@bundeswehr.org (E.M.); markusantwerpen@bundeswehr.org (M.H.A.); romanwoelfel@bundeswehr.org (R.W.); 2 German Centre for Infection Research (DZIF), Partner Site Munich, 80937 Munich, Germany; 3 Central Institute of the Bundeswehr Medical Service Munich, 85784 Garching, Germany; 4 Institute for Infectious Diseases and Zoonoses, Department of Veterinary Sciences, Faculty of Veterinary Medicine, LMU Munich, 80539 Munich, Germany

**Keywords:** variant of concern, omicron, vaccination, hybrid immunity

## Abstract

The SARS-CoV-2 virus has infected more than 660 million people and caused nearly seven million deaths worldwide. During the pandemic, a number of SARS-CoV-2 vaccines were rapidly developed, and several are currently licensed for use in Europe. However, the optimization of vaccination regimens is still ongoing, particularly with regard to booster vaccinations. At the same time, the emergence of new virus variants poses an ongoing challenge to vaccine efficacy. In this study, we focused on a comparative analysis of the neutralization capacity of vaccine-induced antibodies against four different variants of concern (i.e., Alpha, Beta, Delta, and Omicron) after two and three doses of COVID-19 vaccine. We were able to show that both two (prime/boost) and three (prime/boost/boost) vaccinations elicit highly variable levels of neutralizing antibodies. In addition, we did not observe a significant difference in antibody levels after two and three vaccinations. We also observed a significant decrease in the neutralization susceptibility of all but one SARS-CoV-2 variants to vaccine-induced antibodies. In contrast, a SARS-CoV-2 breakthrough infection between the second and third vaccination results in overall higher levels of neutralizing antibodies with a concomitant improved neutralization of all virus variants. Titer levels remained highly variable across the cohort but a common trend was observed. This may be due to the fact that at the time of this study, all licensed vaccines were still based exclusively on wild-type SARS-CoV-2, whereas infections were caused by virus variants. Overall, our data demonstrate the importance of (booster) vaccinations, but at the same time emphasize the need for the continued adaptation of vaccines to induce a protective immune response against virus variants in order to be prepared for future (seasonal) SARS-CoV-2 outbreaks.

## 1. Introduction

The sudden emergence of the severe acute respiratory syndrome coronavirus 2 (SARS-CoV-2) and its rapid global spread led to an exceptionally rapid development of vaccines. Currently, the European Medicines Agency (EMA) has approved six COVID-19 vaccines for use, including two mRNA-based vaccines (Comirnaty/Pfizer-BioNTech and Spikevax/Moderna) and two adenoviral vector-based vaccines (Vaxzevria/AstraZeneca and Jcovden/Janssen-Cilag). In addition, one inactivated (COVID-19 vaccine Valneva/Valneva) and one recombinant vaccine (Nuvaxovid/Novavax) have recently been authorized [1]. With the exception of the inactivated vaccine, which contains inactivated whole virus particles, all other vaccines utilize the spike protein as an antigen [2].

Vaccinations in Germany began in late December 2020 starting with Comirnaty, followed by Spikevax from January 2021 and Vaxzevria from February 2021 [3]. However, in March 2021, the German Immunization Committee restricted the use of Vaxzevria after reports on rare cases of thrombotic events associated with the vaccine [4]. This restriction resulted in many vaccinees receiving a heterologous vaccination when they received either Comirnaty or Spikevax as a second dose [5]. Recent studies have suggested that such a heterologous prime/boost vaccination does indeed induce a stronger immune response with higher antibody levels than a homologous vaccination with vector-based or mRNA-based vaccines [6,7,8,9].

However, further studies have shown that antibody levels decline over time regardless of the vaccination regimen [10,11] and this decline has supported the need for a third vaccine dose to boost the waning immune response. In addition, the almost constant emergence of new virus variants, including several variants of concern (VOCs), has further encouraged the introduction of booster vaccinations worldwide. Nonetheless, vaccine efficacy remains a major concern, as initially licensed vaccines were based exclusively on wild-type SARS-CoV-2 and wild-type spike protein, respectively. However, all present and past VOCs contain various mutations with several mutations found in the spike protein-encoding gene, which may not only increase transmissibility and/or virulence but also enable immune escape [12]. Among the previously described mutations of particular interest in the spike protein rendering VOCs in the literature are amino acid substitutions N501Y, D614G, E484K/A, K417N, and P681H/R [13,14]. Vaccines adapted to variants (such as Comirnaty XBB.1.5) are therefore of great importance to improve the specific immune response, but may take too long to be developed, produced, released, and distributed, creating a constant race between virus and vaccine adaption.

The Alpha variant (lineage B.1.1.7) emerged in the United Kingdom in September 2020 and was named a VOC by the WHO in December 2020. It contains several spike protein mutations and its transmissibility was found to be significantly higher than that of wild-type SARS-CoV-2 [15,16]. The Beta variant (lineage B.1.351) was first identified in South Africa in late 2020 and named a VOC in January 2021. It contains a number of key mutations in the spike protein and it was reported to have a slightly increased transmissibility compared to previously circulating variants [17]. The Delta variant (lineage B.1.617.2) was first identified in India in late 2020 and became the globally dominant strain within just six months, replacing most of the other variants [18]. It was named a VOC in May 2021 by the WHO and contains several mutations in the spike protein, reportedly increasing both transmissibility and immune evasion [17,19,20]. The Omicron variant (lineage B.1.1.529) was first described in South Africa in November 2021 and was named a VOC shortly after [21]. It has more mutations than any previous SARS-CoV-2 variant, many of which are located in the spike protein and more precisely, its receptor-binding domain (RBD) [22]. It has replaced Delta as the globally dominant strain and following the original BA.1 variant, several sub-variants (BA.2-5) have subsequently emerged [23]. Despite its reported increased transmissibility and potential immune escape, Omicron infections show an overall reduced mortality rate compared to Delta [24].

In this study, we investigated the levels of neutralizing antibodies elicited after prime/boost vaccination compared to a third booster vaccination. At the same time, we determined and compared the sensitivity of wild-type SARS-CoV-2 (MUC-IMB-1) and four different VOCs, namely Alpha, Beta, Delta, and Omicron, to neutralization by these antibodies. Lastly, we also examined a small group of individuals, who experienced a confirmed breakthrough infection between their second and third vaccination, and evaluated the infection’s effect on antibody titer and VOC neutralization.

Understanding booster vaccine efficacy and the evolving neutralization efficacy against VOCs is critical for vaccine regimen decisions and potential vaccine adaptations.

## 2. Materials and Methods

### 2.1. Serum Samples

In this study, we analyzed two sets of serum samples. The first subset (n = 56) consisted of samples from individuals who had received their second vaccination against SARS-CoV-2 as part of the prime/boost vaccination scheme. The second subset consisted of samples from individuals who had previously received their third vaccination (n = 62). All samples (n = 118) were collected three weeks (±three days) after vaccination. The majority of the samples tested received a heterologous vaccination, starting with Vaxzevria as the first vaccination and continuing with Comirnaty or Spikevax as the second and third vaccinations, respectively (prime/boost vaccination: 77% heterologous, 23% homologous; prime/boost/boost vaccination: 65% heterologous, 35% homologous). In addition, we analyzed a small subset of samples (n = 10) from individuals who experienced a laboratory-confirmed breakthrough infection between their second and third vaccinations.

All samples evaluated (n = 128) were residual diagnostic material. Therefore, specific information (e.g., demographic characteristics) could not be attributed to individual samples or patients.

### 2.2. Neutralization Test

In-house SARS-CoV-2 neutralization test (NT) was performed as previously described [25]. In brief, serum samples (duplicates) were serially diluted in 96-well tissue culture plates from 1:5 to 1:640. Virus stocks of MUC-IMB-1 (clade B1), B.1.1.7, B.1.351, B.1.617.2, and B.1.351 (50 TCID/50 µL) were prepared using Vero E6 cells and aliquots were stored at −80 °C until use. Each virus was pre-incubated (1 h, 37 °C) with diluted serum samples before Vero E6 cells (104 cells/50 µL) were added to each well. After incubation (72 h, 37 °C), supernatants were discarded and cells were fixed (13% formalin/PBS) and stained (0.1% crystal violet). The neutralizing antibody titer corresponded to the reciprocal of the highest serum dilution showing a complete inhibition of cytopathogenic effects (CPEs). A virus titration control was performed (triplicates) on every plate and exact titers were determined by retrograde calculation. A titer of 5 or greater was considered positive.

### 2.3. Nucleocapsid Protein (NCP) ELISA

All samples were tested for nucleocapsid protein-specific antibodies using a commercial ELISA (Anti-SARS-CoV-2-NCP-ELISA (IgG), EUROIMMUN (Lübeck, Germany)). The ELISA was performed and ratios were calculated according to the manufacturer’s instructions. Results were interpreted as suggested by the manufacturer: ratio < 0.8: negative; ratio ≥ 0.8 to <1.1: borderline; ratio ≥ 1.1: positive.

### 2.4. Isolation and Sequencing of Virus Variants

Replication-competent SARS-CoV-2 viruses were isolated from nasopharyngeal swabs of patients with an RT-qPCR-confirmed SARS-CoV-2 infection. Whole genome sequencing was performed in accordance with the German regulation for molecular genetic surveillance of the coronavirus SARS-CoV-2. Next-generation sequencing and phylogenetic analyses of all viruses used in this study were performed as previously described [26] and lollipop plots were generated using R in combination with the trackViewer library [27]. All viruses were grown on Vero E6 cells. Viral stocks were then prepared, titrated and aliquots were stored at −80 °C until further use.

### 2.5. Statistical Analysis

All statistical analyses (unpaired *t*-test) were performed using GraphPad Prism 8 for Windows (GraphPad Software, San Diego, CA, USA).

## 3. Results

### 3.1. Mutational Changes in Virus Variants

**Figure 1:**
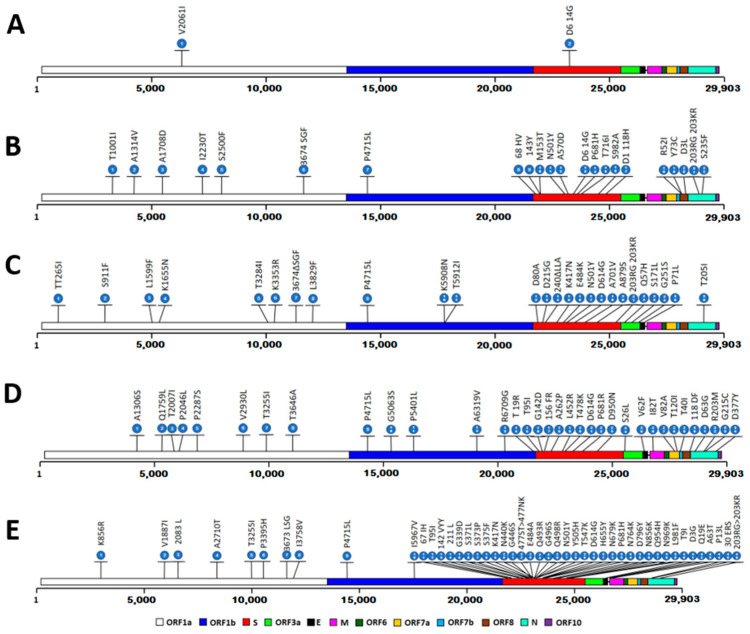
Overview of nonsynonymous mutations within the genomes of the evaluated virus variants of concern (compared to NCBI Ref Seq NC_045512.2) ((**A**): MUC-IMB-1 (**B**): Alpha, (**C**): Beta, (**D**): Delta, and (**E**): Omicron BA.1).

We generated high-quality genomes from patient samples containing replication-competent SARS-CoV-2. Subsequently performed in-depth sequencing analyses revealed various mutations for each isolate relative to the Wuhan sequence, assigning the isolates to lineages B.1.1.7 (Alpha), B.1.617.2 (Delta) and B.1.1.529 (Omicron BA.1). The isolate belonging to lineage B.1.351 (Beta) has been isolated and described in one of our previous studies [28]. Figure 1 gives an overview of all nonsynonymous mutations (compared to the original Wuhan SARS-CoV-2 strain) within the genomes of all four evaluated virus variants as well as MUC-IMB-1. 

In brief, MUC-IMB-1, which serves as the reference strain in this study (hereafter referred to as the wild-type (WT) virus), has two nonsynonymous mutations present in its entire genome, one of which is located within the spike protein (D614G). This is a well-described mutation associated with increased infectivity and fitness [29], which is likely the reason for its epidemiological success and why it is nowadays found in practically all virus variants, including all VOCs evaluated in this study. The Alpha and Beta strains used in this study both contain ten nonsynonymous mutations within the spike protein. However, the only two common mutations are the D614G substitution mentioned above and the N501Y amino acid substitution. Interestingly, the N501Y substitution is also present in Omicron, but not in Delta. The N501Y mutation is located within the receptor binding motif (RBM) of the receptor binding domain (RBD) of the spike protein, which is required for interaction with the angiotensin-converting enzyme 2 (ACE2) of the host receptor. It has previously been shown to enhance infectivity and transmission by increasing the affinity of the viral spike protein for its cellular receptors [30,31].

The Beta strain also contains two other well-described amino acid substitutions associated with increased infectivity, reduced antibody neutralization, and significant immune escape, E484K and K417N, both of which are also located in the ACE2 interaction surface of the spike protein [32,33]. In contrast, Delta also lacks these two mutations, but has gained the P681R substitution, which has previously been described to improve viral fitness by enhancing spike protein cleavage [34]. In addition, Delta has two unique RBD mutations, i.e., T478K and L452R, both associated with increased infectivity and impaired antibody neutralization [35]. Omicron, which has a total of 29 nonsynonymous mutations within its spike protein alone, contains all of the aforementioned mutations (except T478K and L452R) with only two minor substitution variations (i.e., E484A and P681H). Interestingly, the P681H substitution is also present in the Alpha variant and has been shown to enhance spike protein cleavage [36]. Moreover, Omicron has three additional significant mutations within the RBD (i.e., S477N, Q493R, and G496S) all linked to increased affinity of the RBD for the ACE2 and immune escape [37]. Table 1 provides an overview of the occurrence of selected key mutations within the spike protein among all the tested virus variants.

### 3.2. NCP-ELISA

In order not to falsify our results with potential infection-induced antibodies, all sera from vaccinated individuals were initially tested for antibodies against SARS-CoV-2 NCP.

All tested sera (n = 118) were negative for NCP-specific antibodies making a prior infection unlikely. In contrast, all ten sera from the patients with a reported breakthrough infection tested positive for NCP-specific antibodies, confirming the previous SARS-CoV-2 infection (Supplementary Tables S1–S3).

### 3.3. Neutralization Sensitivity of Virus Variants after Prime/Boost Vaccination

**Figure 2:**
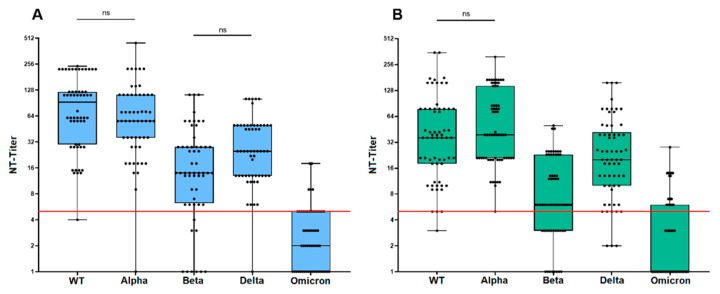
Distribution of NAb titers against all tested variants after two (**A**) and three (**B**) vaccinations. Whiskers extend from minimum to maximum titer levels and all titers are shown Red line indicated the threshold of the NT. Apart from the exceptions indicated (ns), all titer differences between variants were statistically significant. While the neutralization sensitivity of the Alpha variant is largely maintained compared to WT, all other variants show a significant reduction in neutralization sensitivity after two and three vaccinations. The overall lowest titers can be observed against the Omicron variant, followed by the Beta and Delta variant. ((**A**): WT-Alpha: *p* = 0.18, WT-Beta: *p* = 0.0001, WT-Delta: *p* = 0.0001, and WT-Omicron: *p* = 0.0001; Alpha–Beta: *p* = 0.0001, Alpha–Delta: *p* = 0.0001, and Alpha–Omicron: *p* = 0.0001; Beta–Delta: *p* = 0.07 and Beta–Omicron: *p* = 0.0001; Delta–Omicron: *p* = 0.0001). ((**B**): WT-Alpha: *p* = 0.42, WT-Beta: *p* = 0.0001, WT-Delta: *p* = 0.009, and WT-Omicron: *p* = 0.0001; Alpha–Beta: *p* = 0.0001, Alpha–Delta: *p* = 0.0002, and Alpha–Omicron: *p* = 0.0001; Beta–Delta: *p* = 0.0001 and Beta–Omicron: *p* = 0.0001; Delta–Omicron: *p* = 0.0001).

**Figure 3:**
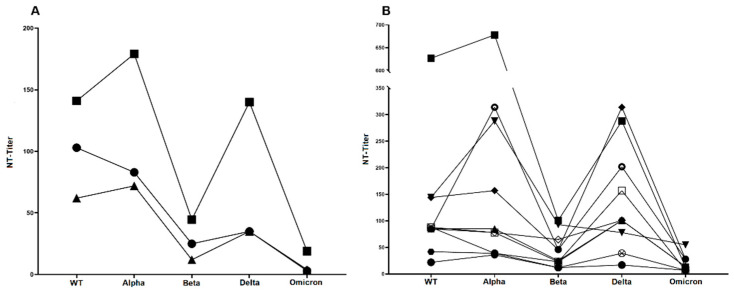
Increased neutralization sensitivity of virus variants after confirmed breakthrough infection. (**A**) Mean NAb titers after two (●) and three (▲) vaccinations and after additional breakthrough infection (▪) reveal overall increased neutralization sensitivity of all virus variants against infection-induced Nab. (**B**) NAb titer developments of all ten triple-vaccinated patients with confirmed breakthrough infection (▲ patient 1, ● patient 2, ▪ patient 3, ▼ patient 4, ⨂ patient 5, ○ patient 6, ⬣ patient 7, ◇ patient 8, ⬥ patient 9, □ patient 10). Although the trends are very similar, the titer levels of the individual patients differ, sometimes considerably. Direct comparison of mean titers after two and three vaccinations reveals statistically significant titer differences for WT titers (103 vs. 62, *p* = 0.0036) and Beta titers (25 vs. 12, *p* = 0.0005).

Figure 2A provides an overview of the distribution of neutralizing antibody (NAb) titers after prime/boost vaccination against each tested variant. Interestingly, NAb titers against each variant were subject to a large variance as indicated by the large span of whiskers. Figure 3A shows the direct comparison of mean titers against the WT virus (MUC-IMB-1) and all four variants revealing an overall decrease in titer levels with the sole exception of the Alpha variant, against which neutralization was largely maintained. However, titer levels against Alpha also showed an exceptionally high variance and when comparing the mean titers, a decrease was also observed. However, this decrease was not significant.

In contrast, the observed decrease in NAb titers against Beta, Delta, and Omicron compared to WT was each statistically significant. Furthermore, we observed a significant decrease in neutralization sensitivity not only compared to WT, but also between the four different variants, except between Beta and Delta. Interestingly, in this case, we observed a slight increase in titers against Delta compared to Beta. However, this increase was not significant. The lowest NAb titers were observed against the Omicron variant, in which 67.9% (38/56) of the sera tested were not able to neutralize. A direct comparison of mean NAb titers to WT (mean titer: 103) and Omicron (mean titer: 3) showed a 32.8-fold reduction in neutralizing sensitivity. The overall decrease in mean titers to the other variants was 1.2 for Alpha (mean titer: 83), 4.1 for Beta (mean titer: 25), and 2.9 for Delta (mean titer: 35) compared to WT (Figure 3A). 

### 3.4. Neutralization Sensitivity of Virus Variants after Third Vaccination

After a third vaccination, the NAb titers against each variant showed a similar variance as after two vaccinations (Figure 2B). As shown in Figure 2B and Figure 3A, (mean) titers against the variants again showed a significant decrease compared to the titers against MUC-IMB-1. An exception was the mean titer against Alpha, which even increased slightly by 1.2-fold (62 vs. 72) compared to the WT titer. However, this increase was not statistically significant. In contrast, the differences in titers between WT and the other variants as well as between the variants were all statistically significant. As with the titers after two vaccinations, there was also a decrease in titers between variants, except for Beta and Delta, which again showed an increase in titers (2.9-fold). However, this 2.9-fold increase was statistically significant in contrast to the double vaccinated group. Omicron titers were also lowest in this group, with 67.7% (42/62) of the sera failing to neutralize Omicron. However, a direct comparison of mean titers to WT (mean titer: 62) and Omicron (mean titer: 4) showed a much smaller decrease of only 15.5-fold. The decrease in mean titers between WT and the other variants was 5.2 for Beta (mean titer: 12) and 1.8 for Delta (mean titer: 35) (Figure 3A).

### 3.5. Neutralization Sensitivity of Virus Variants after Breakthrough Infection

Compared to titers after two and three vaccinations, the mean titers of the triple-vaccinated patients with a confirmed breakthrough infection were significantly higher against all tested variants (Figure 3A). The previously observed decrease in mean titers between WT (mean titer: 140) and variants was also less than in the other two groups and was 3.5 for Beta (mean titer: 40), 1.0 for Delta (mean titer: 140) and only 7.4 for Omicron (mean titer: 19). As observed after three vaccinations, there was also a slight 1.3-fold increase in titers between WT and Alpha. In contrast to Omicron, the differences in titers between WT and the Alpha, Beta, and Delta variants were not statistically significant. The titer differences between Alpha and Delta and Beta and Delta also did not reach statistical significance. Overall, the neutralization sensitivity of Omicron was also the lowest in this group. Thus, Omicron still clearly escaped even infection-induced NAb. Interestingly, there were large differences in titer values between the ten individual patients, while the overall titer trends (i.e., increases and decreases between variants) were very similar (Figure 3B).

### 3.6. Direct Comparison of Neutralization Capacities

**Figure 4:**
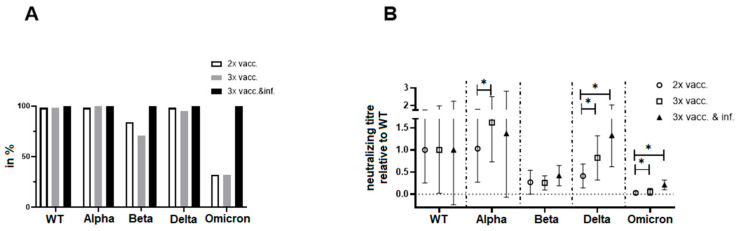
Neutralizing capacity of sera against virus variants. Neutralization of virus variants by sera from patients with different vaccination histories was compared. (**A**) Depicted are the percentages of neutralizing sera (titer ≥ 5) against virus variants for each group. (**B**) Neutralizing titers were normalized to the neutralizing titer against WT by dividing individual titers by the mean titer of WT for every group. The means and standard deviations are depicted. Data were analyzed for multiple comparisons with unpaired *t*-test with Welch correction. Statistically significant differences are marked with * (*p* < 0.05).

The neutralization capacities of sera were lowest for Beta, Omicron, and, to some extent, Delta. However, the neutralization capacities were higher in both the triple-vaccinated and the breakthrough infection groups compared to the double-vaccinated group (Figure 4A). To analyze whether the higher neutralizing capacity after the additional vaccination and/or breakthrough infection is only due to antibody quantity or also antibody quality, the neutralizing titers for each group were normalized to the mean value of the neutralizing titers against WT (Figure 4B). The normalized data again show differences between the double-vaccinated and the other two groups, especially for Delta and Omicron, reflecting the positive effect of repeated antigen contacts. Moreover, there was a trend towards a higher capacity to neutralize VOCs in sera from patients with breakthrough infections compared to samples taken after triple vaccinations. Although not reaching significant levels, this data indicate an improved quality of antibodies induced by hybrid immunity.

## 4. Discussion

In this study, we compared the neutralization sensitivities of SARS-CoV-2 WT and four authentic virus variants to NAb elicited after double and triple COVID-19 vaccination. In addition, we examined the effect of a breakthrough infection between the second and third vaccine doses in a small subset (n = 10) of samples. 

To compare the neutralization sensitivities of the different virus variants, we used an early SARS-CoV-2 isolate (MUC-IMB-1, clade B1) as a reference strain [38]. Compared to the original Wuhan strain sequence, MUC-IMB-1 has two nonsynonymous mutations present in its genome, one of which is a well-described mutation located within the spike protein (D614G). However, this mutation is associated with increased infectivity and fitness [29] and can be found in virtually all SARS-CoV-2 strains, including the variants evaluated in this study. Therefore, the presence of this mutation in our reference strain is unlikely to interfere with our evaluation. 

Overall, we observed a great variation in titer levels against WT virus and virus variants. This underlines the importance of the fact that although NAb levels were generally shown to be predictive of immune protection from symptomatic infection [39], it is important to use currently circulating virus strains when determining NAb to obtain the most accurate and thus, predictive results. This is consistent with the fact that, due to the large variation observed, only limited predictions and assumptions can be made about the neutralization susceptibility of one virus variant based on the results of another strain. 

We found that while both two- and three-vaccination regimens elicited similar overall mean NAb titers, the titer levels in both groups showed a downward trend from WT to Omicron. The only exception was the Alpha variant, for which titers increased slightly by 1.2-fold and 1.3-fold in the triple vaccinees and convalescents, respectively. Interestingly, there was a 1.2-fold decrease in neutralization sensitivity of Alpha in those who had been vaccinated twice. While some studies also described a 2.1- to 2.3-fold decrease in titer levels between WT and the Alpha variant [40,41], others detected a slight increase similar to the ones we observed in triple-vaccinated and convalescent individuals [42]. Overall, the titer increases between WT and Alpha that are visible in our study can only be assessed as secondary, as they are not statistically significant. As the Alpha variant has relatively few mutations mediating immune escape present in its genome compared to other VOCs, a consistent titer level or moderate decrease compared to WT seems most likely and is in line with what we observed. 

Interestingly, we also observed an increase in the neutralization sensitivity of the Delta variant compared to the Beta variant throughout the study, and this observation was independent of the number of vaccinations or breakthrough infections. The increase was significant after triple vaccination (2.9-fold difference) and breakthrough infections (3.5-fold difference), but not after double vaccination. Interestingly, the Delta variant lacks three key mutations in its spike protein, namely amino acid substitutions N501Y, E484K, and K417N, all of which are present in both Beta and Omicron. Both E484K and N501Y are located not only in the RBD but also in the RBM. Several studies [30,40,41,43] have found that all three mutations significantly reduce neutralization sensitivity and drive immune escape, which could explain why Delta shows an improved neutralization sensitivity throughout our study compared to Beta and Omicron. Nonetheless, Delta has two unique mutations responsible for reduced antibody susceptibility, which is in line with our observation that while it is more susceptible to neutralization than Beta and Omicron, it is significantly less susceptible than Alpha, which is missing all of the above-discussed mutations. 

The otherwise overall decrease in titers from WT to Omicron irrespective of vaccination or breakthrough infection is an observation that has been reported by other authors as well [40,44]. However, those studies only evaluated a very limited number of samples, for example, Uriu et al. studied samples from only 14 vaccinated individuals, while Nemet et al. looked at 20 double- and 20 triple-vaccinated individuals. We were able to support these findings with a larger number of patients and samples. In addition, unlike the aforementioned studies, we also performed an NCP-ELISA prior to our evaluation to ensure that no infection-induced antibodies interfered with our study. Nonetheless, it must be mentioned that false negative results of the NCP-ELISA cannot be completely ruled out. 

Not surprisingly, we found that the Omicron variant had the lowest neutralization sensitivity, regardless of the number of vaccinations or breakthrough infections. However, when comparing the neutralizing capacity against virus variants normalized to WT, the benefit of multiple vaccinations and antigen contacts for the functionality of antibodies becomes apparent. This is even more impressive when comparing the percentage of sera that retain any neutralizing capacity against virus variants. In particular, the variants with known escape mutations (Beta and Omicron) show resistance to neutralization by sera from only vaccinated patients. Together with the overall higher titers observed for infection-induced sera, this suggests that the immune response boosted by natural infection (hybrid immunity) is superior to the immune response induced and boosted by vaccination alone and is in agreement with a study by Wilhelm et al. [45]. Interestingly, sera from breakthrough infections show not only the highest neutralizing capacity overall, but show a higher neutralization of WT, Alpha, and Delta than Omicron (despite it most likely being the VOC that has caused the infection). This could be caused by immune imprinting, a mechanism in which the breadth of an immune response triggered by repeated exposure to antigenically closely related but distinct viruses is compromised by pre-existing immunity [46].

In comparison, hybrid immunity likely benefits from a broader immune response based on conserved epitopes. When these epitopes are conserved between the spike protein of the vaccine and the virus variant causing the breakthrough infection, the antibody response to these cross-reactive epitopes is enhanced very efficiently, resembling a heterologous booster immunization [47,48]. In addition, epitopes on proteins other than the spike have previously been shown to be targets of NAbs against coronaviruses. These epitopes are present and recognizable on authentic viruses, but not on spike-based vaccines [49]. They may also promote cross-reactivity, resulting in a higher neutralizing capacity of sera against all variants after a breakthrough infection compared to sera from only vaccinated patients. That these antibodies with new specificities can be induced by breakthrough infections is also shown by the positive results of our NCP ELISA, which detects the nucleocapsid antigen that is not present in the vaccines evaluated in this study Some studies have even suggested an improved durability of hybrid immunity, which may be significantly greater than that provided by repeated vaccinations [50]. Based on the time point of our study and when samples were collected it is also likely that the majority if not all of the ten breakthrough infections we evaluated were caused by Omicron, as it was the dominating VOC at that time. In this respect, it is even surprising, that titers against Omicron were still relatively low. This could be proof of Omicron’s greatly increased capacity to evade immunity, even after natural infection, as previously suggested [51]. Overall, breakthrough infections boosted titers against all VOCs and in contrast to Omicron, titer differences between WT and Alpha, and Beta and Delta variants were not statistically significant, supporting the theory that hybrid immunity promotes cross-reactivity. However, the small group size of only ten sera may have also contributed to the lack of statistical significance, so this result should not be overinterpreted. Our observation that the third vaccination resulted in a smaller titer decline than only two vaccinations may be attributed to the fact that repeated immunization and continued re-exposure to antigens increases antibody affinity due to somatic hypermutation and leads to affinity maturation of the induced antibodies [52]. 

However, our study had several limitations. First, we did not look at the potential effect of non-neutralizing antibodies. Such antibodies are elicited following a viral infection and bind to viruses but seem to be unable to block infection. For this reason, it has long been assumed that they do not play a protective role. However, some studies have suggested the opposite. Fc-mediated effector functions (such as antibody-dependent cellular cytotoxicity or antibody-dependent phagocytosis) were also shown to contribute to protection but were not captured by neutralization assays [53,54].

We were only able to measure a very limited sample size (n = 10) of patients with breakthrough infection (in contrast to the much larger number of samples from vaccinated individuals). In addition, neither disease severity nor comorbidity data were available, both of which may influence titer levels. Factors such as age or gender may also influence the humoral immune response and affect the comparability of titers. However, demographic data were unavailable due to the use of residual diagnostic material. Individual vaccination schedules differed (heterologous vs. homologous), and thus we evaluated a mix of homologous and heterologous vaccinations, which may influence individual results. Nevertheless, our cohorts reflect a significant overall decrease in NAb, regardless of the individual course.

## 5. Conclusions

In conclusion, our data indicate a wide range of NAb titers yet an overall significant decrease in the neutralization susceptibility of all but one SARS-CoV-2 variants to vaccine-induced antibodies compared to WT. We also showed that the specific accumulations of different key mutations within the spike protein of SARS-CoV-2 were associated with a corresponding decrease in the neutralization susceptibility of the respective virus variants. This was particularly striking for the Omicron variant, which showed by far the greatest decline. In addition, a breakthrough infection between the second and third vaccinations was shown to boost the immune response more effectively and as a result, be more beneficial for the neutralization of virus variants than vaccination alone. This was observed for all virus variants, despite the fact that all ten infections were most likely caused by Omicron, potentially highlighting the advantages of hybrid immunity. However, whether this is truly due to hybrid immunization or due to additional contact with viral spike protein cannot be conclusively answered with the dataset of this study.

Taken together, these findings underscore the importance of (booster) vaccination, but also highlight the need to further adapt vaccines to induce a protective immune response against virus variants in order to be prepared for future (seasonal) SARS-CoV-2 outbreaks.

## Figures and Tables

**Table 1:** Overview of key mutations within the spike protein and their occurrence in the virus variants evaluated in this study.

Key Spike Protein Mutations	Location	Described Effect	WT	Alpha	Beta	Delta	Omicron	Reference
D614G	S1 domain (C-terminal)	Enhanced infectivity and fitness	**x**	**x**	**x**	**x**	**x**	[29]
N501Y	Receptor-binding motif	Enhanced infectivity and transmission		**x**	**x**		**x**	[30]
E484K	Receptor-binding motif	Enhanced infectivity, reduced antibody neutralization, and immune escape			x		**x** (E484A)	[32,33]
K417N	Receptor-binding domain	Enhanced infectivity, reduced antibody neutralization, and immune escape			**x**		**x**	[32]
P681R	Furin cleavage site	Enhanced spike protein cleavage and increased fitness		**x** (P681H)		**x**	**x** (P681H)	[34]
T478K	Receptor-binding domain	Enhanced infectivity and reduced antibody neutralization				**x**		[35]
L452R	Receptor-binding domain	Enhanced infectivity and reduced antibody neutralization				**x**		[35]
S477N	Receptor-binding domain	Increased affinity of RBD for ACE2 and immune escape					**x**	[37]
Q493R	Receptor-binding domain	Improved binding between RBD and ACE2					**x**	[37]
G496S	Receptor-binding motif	Reduced antibody-RBD interaction					**x**	[37]

## Data Availability

The data presented in this study are available upon request from the corresponding author. The data are not publicly available due to privacy restrictions.

## Supplementary Materials

The following supporting information can be downloaded at: https://www.mdpi.com/article/10.3390/vaccines12050515/s1, Table S1: Double vaccinated; Table S2: Triple Vaccinated; Table S3: Breakthrough infection.
